# Social comparison orientation and social media fatigue among university students: a cross-sectional study

**DOI:** 10.3389/fpsyg.2026.1861521

**Published:** 2026-09-23

**Authors:** Hala Abd Ellatif Elsayed

**Affiliations:** Department of Health Sciences, College of Health and Rehabilitation, Clinical Psychology Program, Princess Nourah bint Abdulrahman University, Riyadh, Saudi Arabia

**Keywords:** behavioral fatigue, cognitive fatigue, emotional fatigue, social comparison orientation, social media fatigue, university students

## Abstract

Social media platforms frequently expose users to comparison-relevant content, increasing opportunities for continuous self–other evaluation. While social media fatigue has received growing scholarly attention, limited research has examined the direct role of social comparison orientation as an independent factor associated with fatigue. Guided by Social Comparison Theory and a cognitive–affective processing perspective, the present study investigated the association between social comparison orientation and social media fatigue among university students. Using a cross-sectional survey design, data were collected from 601 university students (M_age = 20.20, SD = 2.14; 55.6% female). Participants completed the Iowa–Netherlands Comparison Orientation Measure (INCOM) and the 15-item Social Media Fatigue Scale. Structural equation modeling revealed a strong positive association between social comparison orientation and the higher-order social media fatigue construct (*β* = 0.768, *p* < 0.001), explaining 60.7% of the variance. These findings support the conceptualization of social media fatigue as a multidimensional higher-order construct and indicate that higher levels of social comparison orientation are associated with greater overall social media fatigue among university students. The study contributes to the literature by highlighting social comparison orientation as an important correlate of social media fatigue and advancing the current understanding of fatigue experiences in social media contexts.

## 1 Introduction

Social media platforms have become integral to daily life, particularly among university students, who rely on them for information seeking, social interaction, and self-expression. With the increasing centrality of social media in everyday functioning, scholarly attention has expanded beyond frequency of use to examine the qualitative nature of users’ experiences in social media environments. Recent research has highlighted that prolonged engagement with social media may be associated with subtle yet meaningful psychological outcomes, including cognitive, emotional, and behavioral fatigue (Bright et al., 2015; Dhir et al., 2018; Whelan et al., 2020).

One defining characteristic of contemporary social media platforms is the curation and prioritization of content based on engagement, popularity, and social relevance. These processes tend to amplify comparison-relevant cues, such as peers’ achievements, lifestyles, opinions, and social feedback, thereby increasing users’ exposure to socially evaluative information (Verduyn et al., 2017; Kross et al., 2021). According to Social Comparison Theory, individuals are motivated to evaluate their abilities, opinions, and self-worth through comparisons with others, particularly in contexts where objective standards are absent. In social media environments, opportunities for social comparison are frequent, salient, and often unavoidable.

Importantly, individuals differ in how they attend to and engage with comparison-oriented content on social media. Social comparison has been conceptualized as a relatively stable tendency shaped by individual and contextual factors, including cultural norms and platform affordances (e.g., Vogel et al., 2014). Building on this perspective, these individual differences can be understood as social comparison orientation, defined as a stable dispositional tendency to compare oneself with others across different social contexts (Gibbons and Buunk, 1999; Buunk and Gibbons, 2007). Within social media environments, this general dispositional tendency is expected to influence how individuals attend to, interpret, and respond to comparison-relevant online content. Individuals with stronger social comparison orientation are more likely to engage in sustained evaluative processing, which may shape their emotional and cognitive experiences over time. Consistent with this view, experimental and longitudinal research suggests that exposure to comparison-oriented content on social media promotes sustained self-referential and evaluative processing, which may undermine emotional and cognitive well-being over time (Verduyn et al., 2017).

Empirical studies have shown that excessive exposure to online information is associated with information overload, attentional fatigue, and reduced cognitive efficiency (Eppler and Mengis, 2004; Lee et al., 2016; Li et al., 2023). In addition, repeated engagement in social comparison processes may further intensify self-evaluative cognitive demands (Vogel et al., 2014). Recent evidence among university students supports this processing-based view of fatigue. For example, Niu et al. (2022) found that social media information overload was indirectly associated with adverse outcomes through social fatigue, suggesting that fatigue-related experiences may emerge from sustained cognitive–affective processing demands rather than reflecting usage intensity alone.

Such cumulative cognitive depletion provides a plausible pathway through which comparison-oriented processing may translate into fatigue-related experiences in social media contexts. Cognitive depletion is also likely to extend into the affective and behavioral domains. Cognitive–affective models of self-regulation suggest that sustained cognitive demands can undermine emotional regulation, resulting in irritability, emotional disengagement, and negative affect (Baumeister et al., 2007; Hockey, 2013). In digital contexts, such affective responses may manifest as feelings of annoyance, tension, or emotional exhaustion associated with social media use (Bright et al., 2015; Dhir et al., 2018). Behaviorally, these experiences may translate into reduced motivation to interact, avoidance of posting or reposting content, and tendencies toward disengagement or withdrawal from social media platforms (Whelan et al., 2020).

Collectively, these cognitive, emotional, and behavioral responses have been conceptualized as social media fatigue, a multidimensional construct reflecting users’ exhaustion and disengagement stemming from prolonged, demanding social media experiences. Scale development and validation research has operationalized this construct as comprising three interrelated dimensions—cognitive, emotional, and behavioral fatigue—and supported a refined 15-item measurement model through exploratory and confirmatory factor analyses (Bright et al., 2015; Dhir et al., 2018). This multidimensional framework enables a more nuanced understanding of how fatigue-related experiences emerge across distinct yet interconnected domains of social media use.

Recent meta-analytic evidence indicates that social media fatigue is not simply a function of overall use intensity, but is more strongly associated with specific patterns of engagement. A three-level meta-analysis synthesizing 121 empirical studies found that social media fatigue is strongly associated with problematic and discontinuous use patterns, whereas it is only weakly associated with overall usage frequency (Chen et al., 2023). Complementing these findings, meta-analytic evidence indicates that information overload is associated with social media fatigue, underscoring fatigue as an experiential outcome arising from cumulative cognitive–affective demands during social media use (Ou et al., 2023). These findings highlight the importance of distinguishing fatigue from mere exposure or time spent on social media.

Recent research has conceptualized social media fatigue as an experiential outcome associated with cumulative cognitive and informational demands during social media use. Empirical evidence suggests that exposure to information-rich and socially demanding environments may contribute to fatigue-related experiences, including reduced motivation, irritability, and disengagement from platform use (Bright et al., 2015; Dhir et al., 2018). More recent findings further indicate that information and social overload on digital platforms are associated with social media fatigue and subsequent cognitive depletion, supporting its characterization as an outcome linked to cumulative processing demands rather than merely overall usage intensity (Wang et al., 2025). Taken together, these findings suggest that the psychological consequences of social media use may depend not only on exposure to comparison-relevant content but also on individual differences in how users cognitively engage with comparison-related social information.

Despite extensive research on social comparison processes in social media contexts, existing studies have primarily conceptualized comparison as a user-initiated behavior or a situational emotional response emerging from voluntary engagement with platform content (Vogel et al., 2014; Verduyn et al., 2017). However, contemporary social media environments are characterized by the amplification of socially evaluative and engagement-driven content, thereby increasing repeated exposure to comparison-relevant information (Kross et al., 2021; Serttaş et al., 2025). Moreover, social comparison has been linked to fatigue-related experiences in social media environments. Prior research has shown that comparison-oriented processing contributes to cognitive and emotional demands associated with platform use, which may culminate in social media fatigue (Cramer et al., 2016; Niu et al., 2022). Although previous studies have linked social comparison with fatigue-related experiences on social media, several important gaps remain. First, much of the existing literature has focused on comparison as a situational behavior or specific comparison experiences rather than on social comparison orientation as a stable dispositional tendency. Second, relatively few studies have examined social media fatigue as a multidimensional higher-order construct encompassing cognitive, emotional, and behavioral dimensions. Third, empirical evidence from Saudi university students remains limited. Addressing these gaps, the present study examines the association between dispositional social comparison orientation and multidimensional social media fatigue among Saudi university students using a higher-order structural equation modeling framework.

### 1.1 Theoretical framework

This study is grounded in Social Comparison Theory, which posits that individuals evaluate their abilities, opinions, and self-worth through comparisons with others, particularly in the absence of objective standards. Extensive research has demonstrated that social comparison processes are cognitively effortful and involve continuous self-evaluation and interpretation of social information (Festinger, 1954; Wood, 1989; Buunk and Gibbons, 2007). Within digital environments, these processes are intensified because platforms systematically highlight comparison-relevant cues such as achievements, lifestyles, and social feedback, thereby increasing opportunities for continuous self–other evaluation and sustained evaluative processing (Vogel et al., 2014; Verduyn et al., 2017).

Early empirical work has linked social comparison processes on social networking sites to experiences of social media fatigue. For example, Cramer et al. (2016) showed that frequent engagement in social comparison on Facebook was associated with affective consequences and a growing desire to disengage from the platform, a phenomenon referred to as Facebook fatigue. More recent research has further emphasized that social comparisons on social media involve sustained attentional and evaluative processing that shapes users’ cognitive and emotional experiences during platform use. Consistent with this perspective, subsequent studies have demonstrated that social comparison processes play a central role in the experience of social media fatigue. For instance, Jabeen et al. (2023) reported that social comparison on social networking sites was positively associated with social media fatigue, highlighting the cognitively and emotionally demanding nature of sustained comparison during platform use.

Importantly, social media comparisons are not solely the result of users’ active choices but are also influenced by platform content dynamics. Content that is socially salient and engaging often contains implicit or explicit comparison signals, leading to repeated exposure to comparison-relevant information (Kross et al., 2021). Within social media environments, social comparison may therefore assume a more persistent cognitive character rather than remaining an occasional evaluative activity. In this context, social comparison orientation reflects individuals’ tendency to attend to, engage with, and cognitively elaborate on comparison-related content during social media use. This orientation represents a general tendency to compare oneself with others across contexts, rather than a context-specific form of comparison.

In addition, social comparison orientation extends beyond transient comparison behaviors or momentary emotional reactions. Drawing on social information-processing perspectives (Crick and Dodge, 1994; Lemerise and Arsenio, 2000), this tendency can be conceptualized as a relatively stable cognitive–affective processing pattern that shapes how individuals attend to, interpret, and elaborate comparison-relevant information. Prior research on social comparison orientation has similarly emphasized stable individual differences in comparison-related processing (Gibbons and Buunk, 1999; Buunk and Gibbons, 2007). Within social media environments, such stable processing tendencies may intensify exposure-related cognitive demands, potentially contributing to fatigue-related experiences during social media use (Bright et al., 2015; Dhir et al., 2018).

Social media environments shape the context in which social comparison occurs by prioritizing salient and engagement-driven content that embeds implicit and explicit evaluative cues (Metzler and Garcia, 2024). Through engagement optimization and feedback loops between user behavior and platform design, such environments may amplify exposure to comparison-relevant material and increase opportunities for social comparison across digital interactions (Fardouly et al., 2015). Repeated exposure to idealized and socially evaluative content has been linked to heightened self-evaluative processing and negative affective responses, suggesting that comparison experiences may become more persistent and cognitively elaborative over time (Vogel et al., 2014; Kross et al., 2021). Collectively, these findings indicate that such processes may not merely increase comparison frequency but may also shape the qualitative nature of comparison experiences within digital contexts.

From a cognitive load perspective, repeated exposure to comparison-rich information imposes sustained attentional and evaluative demands that may exceed available cognitive resources (Eppler and Mengis, 2004; Lee et al., 2016). Over time, these cumulative processing demands may manifest as multidimensional social media fatigue encompassing cognitive depletion, emotional exhaustion, and behavioral disengagement (Bright et al., 2015; Dhir et al., 2018; Wang et al., 2023). Accordingly, such amplification processes provide a theoretically grounded mechanism through which comparison-oriented processing may be associated with fatigue-related experiences in social media environments.

To explain how such orientation translates into psychological outcomes, the present study adopts a cognitive–affective processing perspective grounded in established models of information processing. Social information processing theories emphasize that individuals differ in how they selectively attend to, interpret, and respond emotionally to social cues, and that these processing tendencies shape subsequent outcomes (Crick and Dodge, 1994; Lemerise and Arsenio, 2000). In social media use, comparison-oriented processing requires sustained attentional allocation, continuous evaluative judgment, and the integration of self-relevant information, all of which are likely to involve increased cognitive demands.

Consistent with Cognitive Load Theory, cognitive resources are finite, and repeated engagement in demanding evaluative processing may be associated with cumulative cognitive depletion (Sweller, 1988). Prior research has shown that exposure to excessive or socially salient online information is associated with information overload, attentional fatigue, and reduced cognitive capacity, even when users do not consciously perceive the experience as stressful (Eppler and Mengis, 2004; Lee et al., 2016; Whelan et al., 2020; Dai et al., 2024). Thus, individuals with higher social comparison orientation may be more likely to experience greater cognitive fatigue during sustained processing of comparison-rich content.

Beyond cognitive consequences, cognitive depletion may extend into the affective domain. Cognitive–affective models suggest that prolonged cognitive demands can reduce emotional regulation capacity, resulting in irritability, tension, and emotional disengagement (Baumeister et al., 2007; Hockey, 2013). In digital contexts, such affective responses have been conceptualized as social media fatigue, reflecting a state of emotional weariness and negative affect associated with continued platform use (Bright et al., 2015; Dhir et al., 2018). Importantly, this fatigue does not require conscious perceptions of stress but may emerge gradually through sustained cognitive–affective processing demands.

Empirical evidence suggests that information-rich social media environments intensify social comparison processes, which in turn contribute to fatigue-related experiences. For example, negative social comparison and fatigue from Social Networking Sites (SNS) have been shown to jointly mediate the association between information overload and intentions to reduce SNS usage (Niu et al., 2020). Consistent with this perspective, social comparison behaviors have been positively associated with social media fatigue (Jabeen et al., 2023), while broader cognitive load frameworks highlight comparison-oriented processing as a theoretically relevant contributor to fatigue-related experiences in digital contexts (Qin et al., 2024). Moreover, recent findings indicate that upward and parallel social comparisons are positively associated with social media fatigue and related psychosocial outcomes (Zhu and Yao, 2025).

Taken together, prior research suggests that social media environments amplify comparison-relevant cues and may intensify comparison-oriented cognitive–affective processing. However, existing studies have primarily examined social comparison as a situational behavior or emotional response rather than as a stable processing tendency. Building on Social Comparison Theory and cognitive–affective processing perspectives, the present study conceptualizes social comparison orientation as a stable individual difference that reflects users’ propensity to attend to and elaborate on comparison-relevant information during social media use. From this perspective, individuals with a higher social comparison orientation may experience greater multidimensional social media fatigue due to sustained demands for evaluative processing.

### 1.2 Research hypotheses

Based on Social Comparison Theory and the conceptualization of social media fatigue as a higher-order construct, the following hypothesis was formulated:

*H1*: Higher levels of social comparison orientation are expected to be positively associated with overall social media fatigue among university students.

## 2 Methods

### 2.1 Study design and participants

The present study employed a quantitative cross-sectional survey design to examine the association between social comparison orientation and social media fatigue among university students.

A convenience sampling strategy was adopted using online recruitment. The survey link was distributed among undergraduate and postgraduate students across multiple universities in Saudi Arabia through institutional communication channels and voluntary sharing. The host university also facilitated the dissemination of the questionnaire to other universities, enhancing the diversity of the sample. Eligibility criteria included being 18 years of age or older, current enrollment in a Saudi university, current use of at least one social media platform, and provision of informed consent. Participants were not asked to identify the specific university they attended; therefore, information regarding the participating universities and the distribution of participants across institutions was not collected.

Participation was voluntary, and informed consent was obtained electronically prior to data collection. Ethical approval was granted by the Institutional Review Board of a university in Saudi Arabia (IRB Log No. 26–0274). All procedures were conducted in accordance with the ethical standards for research involving human participants and the principles of the Declaration of Helsinki.

### 2.2 Measures

#### 2.2.1 Social comparison orientation

Social comparison orientation was assessed using the Iowa–Netherlands Comparison Orientation Measure (INCOM) originally developed by Gibbons and Buunk (1999). An Arabic version of the scale was used in the present study. Permission to use the Arabic instrument was obtained from the researcher who previously employed the scale in Arabic research contexts (Al-Lihyani, 2020). Participants rated their agreement with each statement using a five-point Likert scale ranging from 1 (strongly disagree) to 5 (strongly agree), with higher scores indicating stronger social comparison orientation.

As part of the confirmatory factor analysis, the two reverse-worded INCOM items (Items 6 and 10) demonstrated comparatively weaker psychometric performance than the remaining items and were therefore excluded from the final measurement model. Because both items were reverse-scored, their weaker performance may partly reflect method effects associated with reverse wording and/or cross-linguistic adaptation rather than theoretical irrelevance to the construct. The decision to exclude these items was therefore treated as a sample-specific psychometric refinement rather than a modification of the theoretical definition of social comparison orientation. This approach is consistent with previous psychometric research indicating that the original INCOM item structure may require refinement across different samples and cultural adaptations (Schneider and Schupp, 2011; Veleda et al., 2024). The nine-item specification demonstrated acceptable internal consistency (Cronbach’s α = 0.74; McDonald’s ω = 0.74).

#### 2.2.2 Social media fatigue

Social media fatigue was measured using the 15-item Social Media Fatigue Scale developed by Zhang et al. (2016), which conceptualizes fatigue as a multidimensional construct comprising three interrelated dimensions: cognitive, behavioral, and emotional fatigue.

Because the original instrument was developed in English, the scale was translated into Arabic using a standard translation–back translation procedure. First, the items were translated into Arabic by a bilingual researcher with expertise in psychology. The Arabic version was then independently back-translated into English by another bilingual translator who was not involved in the initial translation. The two English versions were compared to ensure conceptual equivalence, and minor wording adjustments were made where necessary.

Participants responded using a five-point Likert scale ranging from 1 (strongly disagree) to 5 (strongly agree). To examine the psychometric adequacy of the Arabic version of the 15-item Social Media Fatigue Scale, a Confirmatory Factor Analysis (CFA) was conducted using the 15 scale items only. Three competing measurement models were evaluated: a one-factor model, a correlated three-factor model, and a higher-order model. The one-factor model demonstrated comparatively poorer fit (χ^2^(90) = 179.06, CFI = 0.943, TLI = 0.933, RMSEA = 0.045, SRMR = 0.040). Both the correlated three-factor model (χ^2^(87) = 164.24, CFI = 0.951, TLI = 0.940, RMSEA = 0.042, SRMR = 0.039) and the higher-order model (χ^2^(87) = 164.24, CFI = 0.951, TLI = 0.940, RMSEA = 0.042, SRMR = 0.039) demonstrated acceptable model fit. The higher-order model was retained for subsequent analyses because it is consistent with the theoretical conceptualization of social media fatigue as a higher-order construct. The internal consistency coefficients were acceptable, with Cronbach’s alpha values of 0.64 for cognitive fatigue, 0.70 for behavioral fatigue, and 0.70 for emotional fatigue, and McDonald’s omega values ranging from 0.64 to 0.70, supporting the scale’s reliability.

### 2.3 Procedure

Data were collected through an online questionnaire administered via Google Forms. After accessing the survey link, participants first reviewed an informed consent statement that explained the study’s purpose, the voluntary nature of participation, and the confidentiality of responses. Participants who agreed to participate completed the questionnaire.

The survey link was disseminated electronically through the Vice Presidency for Research at Princess Nourah bint Abdulrahman University, which coordinated its distribution to university students across Saudi universities through official academic communication channels.

The survey included demographic questions followed by the measures of social comparison orientation and social media fatigue. No personally identifying information was collected, and all responses were recorded anonymously. Completing the survey took approximately 8–10 min. During data screening, straight-lining responses were operationally defined as responses in which participants selected the same response category for every item. Such cases were excluded from the final dataset.

### 2.4 Data analysis

Before proceeding to the primary analyses, participant flow and data quality screening procedures were conducted. A total of 654 responses were initially received through the online survey. Of these, 26 incomplete questionnaires were excluded, leaving 628 eligible responses for further screening. Subsequently, five cases were excluded for not meeting the age eligibility criterion, and 11 cases exhibiting straight-lining response patterns indicative of low-quality responses were removed. Finally, multivariate outliers were identified using the Mahalanobis distance (D^2^). Based on a χ^2^ distribution with 24 degrees of freedom and a conservative threshold of *p* < 0.001 (Tabachnick and Fidell, 2019), 11 multivariate outliers were excluded, resulting in a final analytical sample of *N* = 601.

Quantitative data analyses were conducted using Rstudio (Version 2026.01.1). The analytical process followed three main stages. First, the dataset was evaluated to ensure its suitability for structural equation modeling. Univariate normality was assessed by examining the skewness and kurtosis of each item. Data were considered to follow a univariate normal distribution when skewness values were within ±3, and kurtosis values were within ±10 (Kline, 2023). As shown in Table 1, all items fell well within these parameters. Multicollinearity was examined using the Variance Inflation Factor (VIF). VIF values ranged from 1.19 to 1.55, remaining well below the commonly recommended threshold of 5.0 (Hair et al., 2019), indicating that multicollinearity did not threaten model stability. Common method bias (CMB) was assessed using Harman’s single-factor test. An unrotated principal component analysis indicated that the first factor accounted for 26.64% of the total variance, which is below the 50% threshold recommended by Podsakoff et al. (2003).

**Table 1:** Data screening diagnostics: normality and multicollinearity (*N* = 601).

Items	Skewness	Kurtosis	VIF
SCO_1	−0.17	−0.94	1.24
SCO_2	0.11	−1.01	1.31
SCO_3	0.11	−1.08	1.30
SCO_4	0.13	−1.06	1.28
SCO_5	0.12	−1.06	1.30
SCO_7	0.00	−1.16	1.29
SCO_8	0.07	−1.07	1.19
SCO_9	0.05	−1.05	1.36
SCO_11	−0.11	−0.97	1.38
SMF_1	−0.12	−1.13	1.28
SMF_2	0.20	−1.11	1.39
SMF_3	0.36	−0.93	1.37
SMF_4	0.40	−0.99	1.39
SMF_5	0.31	−1.08	1.40
SMF_6	0.43	−0.83	1.42
SMF_7	0.39	−0.97	1.45
SMF_8	0.41	−0.97	1.55
SMF_9	0.47	−0.85	1.45
SMF_10	0.31	−1.08	1.44
SMF_11	0.29	−0.98	1.42
SMF_12	0.32	−1.02	1.42
SMF_13	0.49	−0.78	1.45
SMF_14	0.54	−0.73	1.40
SMF_15	0.31	−1.00	1.39

SCO is Social Comparison Orientation. SMF is Social Media Fatigue.

Second, the measurement model was evaluated using Confirmatory Factor Analysis (CFA) with the robust Maximum Likelihood estimator (MLR) implemented through the lavaan package in R. Model fit was assessed using widely recommended indices, including the Comparative Fit Index (CFI), Tucker–Lewis Index (TLI), Root Mean Square Error of Approximation (RMSEA), and Standardized Root Mean Square Residual (SRMR).

Finally, Structural Equation Modeling (SEM) was employed to test the hypothesized relationships between social comparison orientation and social media fatigue. A higher-order structural model was estimated, with age, gender, academic level, and academic major included as control variables. Statistical significance was evaluated at *p* < 0.05.

### 2.5 Ethical considerations

The study protocol was reviewed and approved by the Institutional Review Board (IRB) of a university in Saudi Arabia (IRB Log No. 26–0274; approval date: February 4, 2026). The study was classified as minimal risk and received expedited approval. All participants were informed about the purpose of the study, the voluntary nature of participation, and their right to withdraw at any time. Informed consent was obtained electronically prior to participation, and all responses were collected anonymously in accordance with the ethical principles outlined in the Declaration of Helsinki.

## 3 Results

### 3.1 Measurement model evaluation

To evaluate the higher-order measurement model used in the subsequent structural analyses, a Confirmatory Factor Analysis (CFA) was conducted using the robust Maximum Likelihood estimator (MLR) in R (lavaan package). Social Media Fatigue was specified as a higher-order construct reflected by the cognitive, behavioral, and emotional dimensions. The higher-order measurement model yielded excellent fit indices: *X*^2^(248) = 360.07, *p* < 0.001, Robust Comparative Fit Index (CFI) = 0.955, Robust Tucker-Lewis Index (TLI) = 0.950, Robust Root Mean Square Error of Approximation (RMSEA) = 0.029, and Standardized Root Mean Square Residual (SRMR) = 0.037. All fit statistics exceeded the recommended thresholds (CFI and TLI > 0.90; RMSEA and SRMR < 0.08) suggested by Hu and Bentler (1999).

To further examine the factorial structure of the Social Media Fatigue construct, alternative measurement models were also estimated and compared, including a one-factor model, a correlated three-factor model, and a higher-order model. The one-factor model demonstrated the weakest fit to the data (χ^2^ = 179.06, df = 90, CFI = 0.943, TLI = 0.933, RMSEA = 0.045, SRMR = 0.040). In contrast, both the correlated three-factor model and the higher-order model demonstrated identical fit, outperforming the one-factor model (χ^2^ = 164.24, df = 87, CFI = 0.951, TLI = 0.940, RMSEA = 0.042, SRMR = 0.039). No estimation warnings or boundary estimates were encountered during model estimation. Given the equivalent fit of the correlated three-factor and higher-order models, the higher-order model was retained for the subsequent structural analyses based on theoretical considerations, as it is consistent with the conceptualization of social media fatigue as a multidimensional higher-order construct.

#### 3.1.1 Reliability and validity analysis

As shown in Table 2, the evaluation of the measurement model was conducted using a dual-focus approach that included internal consistency reliability and convergent validity. Regarding reliability, the results indicated that the constructs possess adequate internal consistency. The Cronbach’s alpha (α) and McDonald’s omega (ω) values for Social Comparison Orientation (SCO) were 0.735, above the conventional threshold of 0.70. The dimensions of Social Media Fatigue (Cognitive, Behavioral, and Emotional) also demonstrated acceptable coefficients, with Cronbach’s alpha and McDonald’s omega values ranging from 0.640 to 0.690, indicating acceptable albeit modest internal consistency for these subscales. Furthermore, convergent validity was evaluated using standardized factor loadings and the Average Variance Extracted (AVE). All item loadings were statistically significant at *p* < 0.001, with standardized loadings ranging from 0.400 to 0.602. Although several loadings were relatively modest, all exceeded the minimum recommended threshold of 0.40. Additionally, the second-order factor (Global SMF) showed high standardized loadings on its first-order dimensions: Cognitive (0.966), Behavioral (0.999), and Emotional (0.900), supporting the higher-order structure (Figure 1). Although the AVE values were below the conventional threshold of 0.50, the evidence for convergent validity should be interpreted with caution, and further validation across different samples and cultural contexts is warranted.

**Table 2:** Standardized factor loadings, reliability, and convergent validity.

Construct	Items	Std. loading	α	McDonald’s omega (ω)	AVE
SCO	SCO_1	0.400	0.735	0.735	0.238
	SCO_2	0.485			
	SCO_3	0.497			
	SCO_4	0.468			
	SCO_5	0.496			
	SCO_7	0.496			
	SCO_8	0.416			
	SCO_9	0.555			
	SCO_11	0.551			
Social media fatigue (second-order factor)					
Cognitive fatigue (reflected loading: 0.966)	SMF_1	0.433	0.640	0.640	0.263
	SMF_2	0.534			
	SMF_3	0.533			
	SMF_4	0.521			
	SMF_5	0.540			
Behavioral fatigue (reflected loading: 0.999)	SMF_6	0.522	0.690	0.700	0.315
	SMF_7	0.560			
	SMF_8	0.602			
	SMF_9	0.576			
	SMF_10	0.542			
Emotional fatigue (reflected loading: 0.900)	SMF_11	0.546	0.690	0.690	0.313
	SMF_12	0.556			
	SMF_13	0.582			
	SMF_14	0.564			
	SMF_15	0.550			

**Figure 1:**
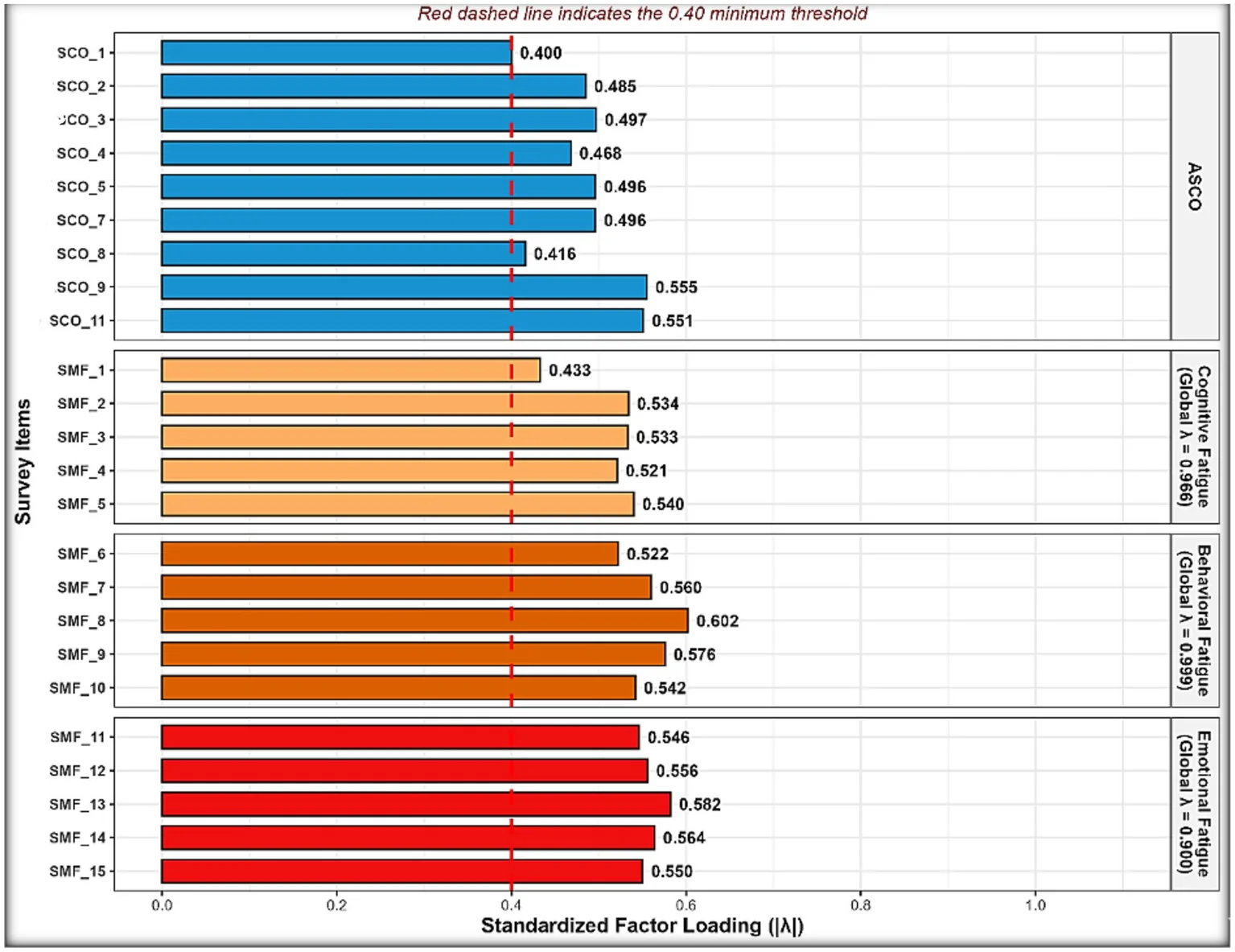
Standardized factor loading for the measurement model. All items exceed the minimum threshold of 0.40 for convergent validity.

N = 601. The two reverse-worded INCOM items (Items 6 and 10) were excluded during model refinement. SMF dimensions were modeled as a second-order latent construct (Global SMF). Std. Loading = Standardized Factor Loading (represents the correlation between the item and its latent construct); α = Cronbach’s Alpha (coefficient of internal consistency); (ω) = McDonald’s omega (coefficient of internal consistency reliability). All factor loadings were statistically significant at *p* < 0.001.

#### 3.1.2 Discriminant validity

The high correlations among the cognitive, emotional, and behavioral dimensions indicate substantial overlap among these dimensions in the present sample. Although this pattern is consistent with the theoretical conceptualization of social media fatigue as a higher-order construct, it also suggests that the empirical distinctiveness of the three dimensions was limited in the current sample. Accordingly, the higher-order model should be interpreted as reflecting closely related manifestations of a common underlying construct rather than as strongly differentiated dimensions within the present sample (Table 3).

**Table 3:** Latent variable correlations (discriminant validity).

Construct	1	2	3	4
1. SCO	(0.488)			
2. Cognitive fatigue	0.750	(0.513)		
3. Behavioral fatigue	0.775	0.964	(0.561)	
4. Emotional fatigue	0.699	0.869	0.899	(0.560)

Values in parentheses along the diagonal represent the square root of the Average Variance Extracted (AVE). Off-diagonal values represent latent variable correlations (Fornell and Larcker, 1981). The high correlations among the cognitive, behavioral, and emotional dimensions are consistent with their conceptualization as closely related dimensions of social media fatigue.

### 3.2 Participant characteristics

The demographic profile of the final sample (N = 601) is presented in Table 4. The participants’ ages ranged from 18 to 45 years, with a mean age of 20.20 years (SD = 2.14). Although the sample was predominantly composed of traditional university-aged students, a small proportion of older students was also included, resulting in a wider age range. The sample consisted of females (55.6%) and males (44.4%). The vast majority was single (86.9%). Regarding academic levels, almost half of the sample were first-year students (47.1%), followed by second-year students (33.4%). The participants represented diverse academic backgrounds; students were distributed across Natural Sciences (19.3%), Health/Medical Sciences (16.6%), and Humanities/Social Sciences (15.6%), while the remaining participants (48.4%) were classified in the “Other” category. Accordingly, for analytical purposes, academic majors were grouped into four broad disciplinary categories: Natural Sciences, Health/Medical Sciences, Humanities/Social Sciences, and Other. The “Other” category included academic majors such as Law, Agriculture, Business Administration, Engineering, and Computer Science, which did not fall within the first three predefined categories.

**Table 4:** Demographic characteristics of the participants (*N* = 601).

Variable	Category	Frequency (n)	Percentage (%)
Gender	Female	334	55.6
	Male	267	44.4
Academic level	First year	283	47.1
	Second year	201	33.4
	Third year	28	4.7
	Fourth year	69	11.5
	Fifth year	13	2.2
	Postgraduate	7	1.2
Academic major	Natural sciences	116	19.3
	Health/medical sciences	100	16.6
	Humanities/social sciences	94	15.6
	Other	291	48.4
Marital status	Single	522	86.9
	Married	45	7.5
	Other	34	5.7

Age is a continuous variable: Mean = 20.20, SD = 2.14, Min = 18, Max = 45.

### 3.3 Item-level and construct-level descriptives

To evaluate the participants’ responses across the studied constructs, descriptive statistics, including means, standard deviations, and the Relative Importance Index (RII), were calculated. As shown in Figure 2, the RII values for the Social Comparison Orientation (SCO) items ranged from 57.67 to 63.43%, indicating a moderate-to-high tendency toward social comparison among the students. Similarly, Social Media Fatigue (SMF) items displayed RII values ranging from 54.14 to 65.49%.

**Figure 2:**
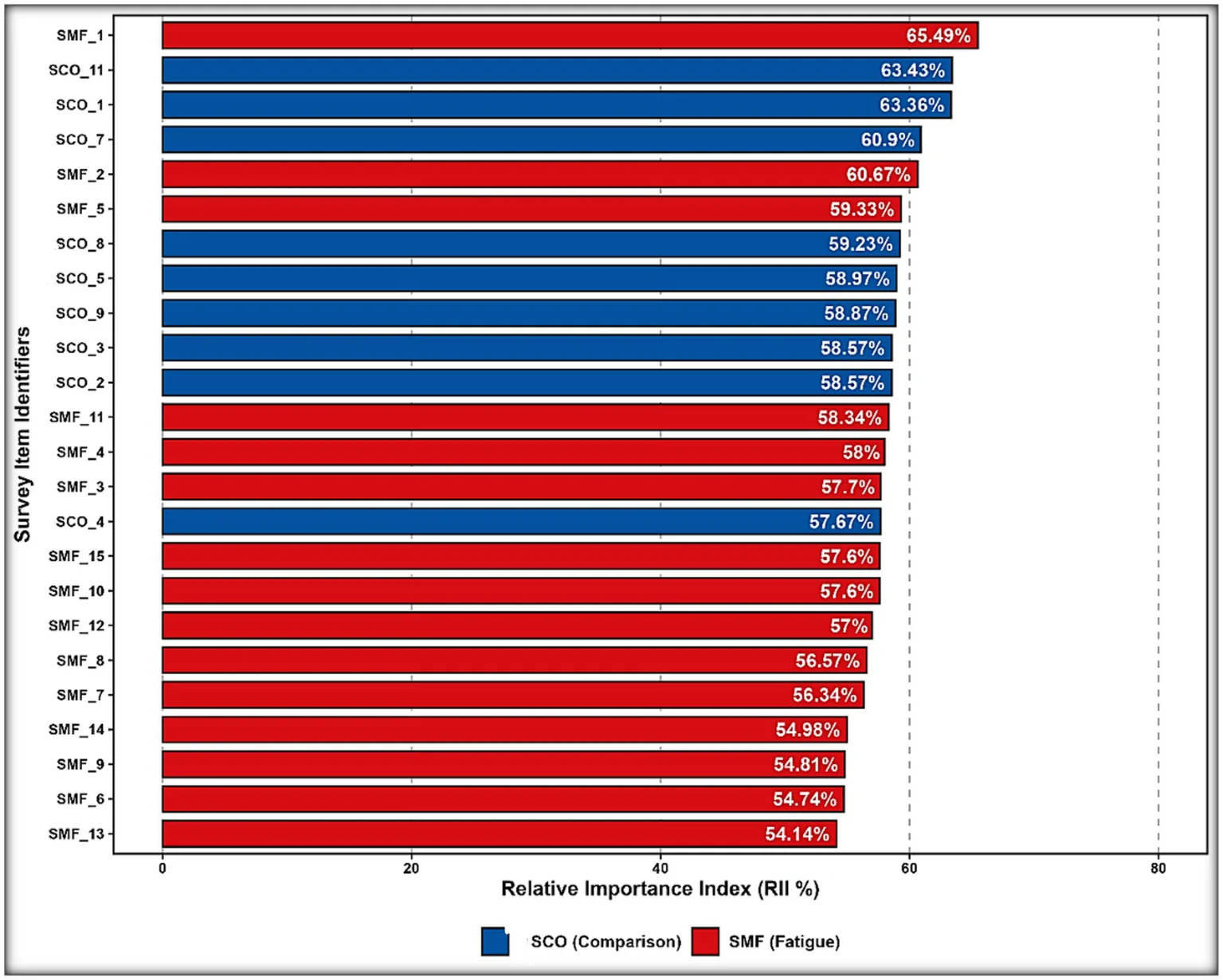
Item-level analysis and relative importance index (RII).

Furthermore, composite scores were calculated for each main construct and its sub-dimensions (Table 5). The overall SCO score was moderate (M = 3.00, SD = 0.71). For Social Media Fatigue, Cognitive Fatigue emerged as the most prominent dimension (M = 3.01, SD = 0.78), followed by Emotional Fatigue (M = 2.82, SD = 0.81) and Behavioral Fatigue (M = 2.80, SD = 0.83). The overall SMF score was also moderate (M = 2.88, SD = 0.70).

**Table 5:** Descriptive statistics for main constructs and sub-dimensions.

Construct/dimension	M	SD	Level
Social media fatigue (total)	2.88	0.70	Moderate
Cognitive fatigue	3.01	0.78	Moderate
Emotional fatigue	2.82	0.81	Moderate
Behavioral fatigue	2.80	0.83	Moderate
Social comparison orientation (total)	3.00	0.71	Moderate

Scores are based on a 5-point Likert scale.

### 3.4 Bivariate correlation analysis

Prior to testing the structural models, Pearson correlation coefficients were calculated to examine the initial bivariate relationships among the composite variables: Social Comparison Orientation (SCO), the three dimensions of Social Media Fatigue (SMF), and Overall SMF (Figure 3). All relationships among the study variables were positive and statistically significant (*p* < 0.001). Specifically, SCO demonstrated a stronger positive correlation with Overall SMF (*r* = 0.604). SCO also showed the strongest correlation with Behavioral Fatigue (*r* = 0.552), followed closely by Cognitive Fatigue (*r* = 0.539), and Emotional Fatigue (*r* = 0.476). Furthermore, the internal dimensions of SMF were highly correlated with each other (ranging from *r* = 0.591 to *r* = 0.639) and with the overall SMF construct (ranging from *r* = 0.852 to *r* = 0.880).

**Figure 3:**
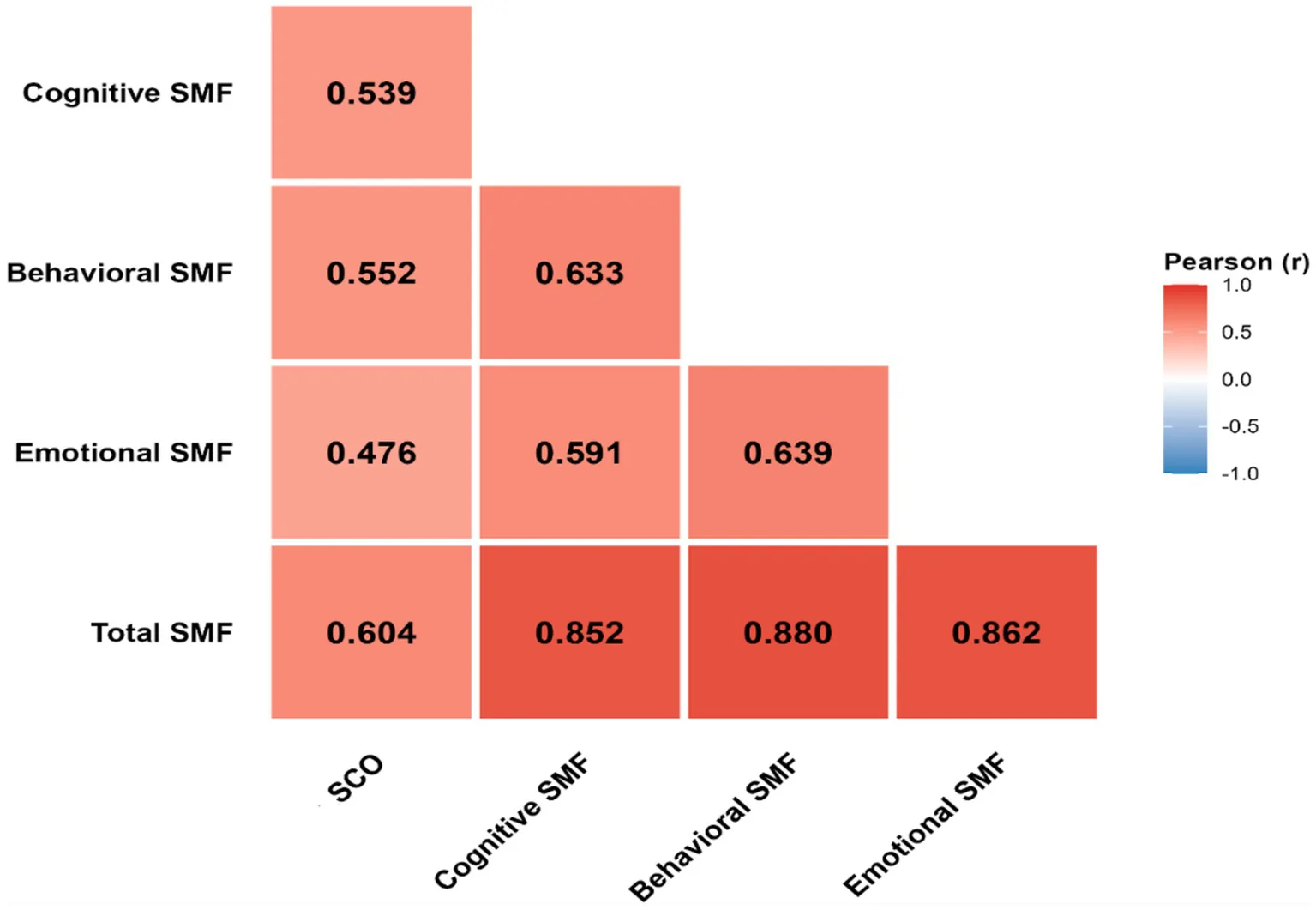
Pearson correlation heatmap of the study’s main variables and sub-dimensions. All correlations are statistically significant at *p* < 0.001.

### 3.5 Structural equation modeling (SEM) and hypothesis testing

To test the primary hypothesis after controlling for demographic variables (Age, Gender, Academic Level, and Academic Major), a Higher-Order Structural Equation Modeling (SEM) with a robust Maximum Likelihood (MLR) estimator was employed. This model tested the direct effect of Social Comparison Orientation (SCO) on the higher-order Social Media Fatigue (SMF) construct, reflected in its cognitive, behavioral, and emotional dimensions. Because social media fatigue was conceptualized and modeled as a higher-order construct, the hypothesized association was evaluated at the level of the global latent factor. Accordingly, the cognitive, emotional, and behavioral dimensions were modeled as first-order factors reflecting the higher-order social media fatigue construct rather than as separate structural outcome variables.

The higher-order structural model demonstrated an excellent fit to the data: χ^2^(386) = 598.74, *p* < 0.001; Robust CFI = 0.923; Robust TLI = 0.916; Robust RMSEA = 0.031; and SRMR = 0.043. The results supported the primary hypothesis, revealing a positive and statistically significant association between SCO and overall SMF (β = 0.768, *p* < 0.001). The inclusion of SCO, along with the demographic controls, explained 60.7% of the variance (R^2^ = 0.607) in overall social media fatigue.

Furthermore, the higher-order SMF construct was strongly reflected by its three first-order dimensions, explaining 94.4% of the variance in Cognitive Fatigue, 98.6% in Behavioral Fatigue, and 80.6% in Emotional Fatigue. These findings indicate that the association between social comparison orientation and social media fatigue is reflected through its interrelated cognitive, emotional, and behavioral dimensions.

Regarding the control variables, none of the demographic factors (Age, Gender, Academic Level, or Major) showed significant effects (*p* > 0.05) (Figure 4, Table 6).

**Figure 4:**
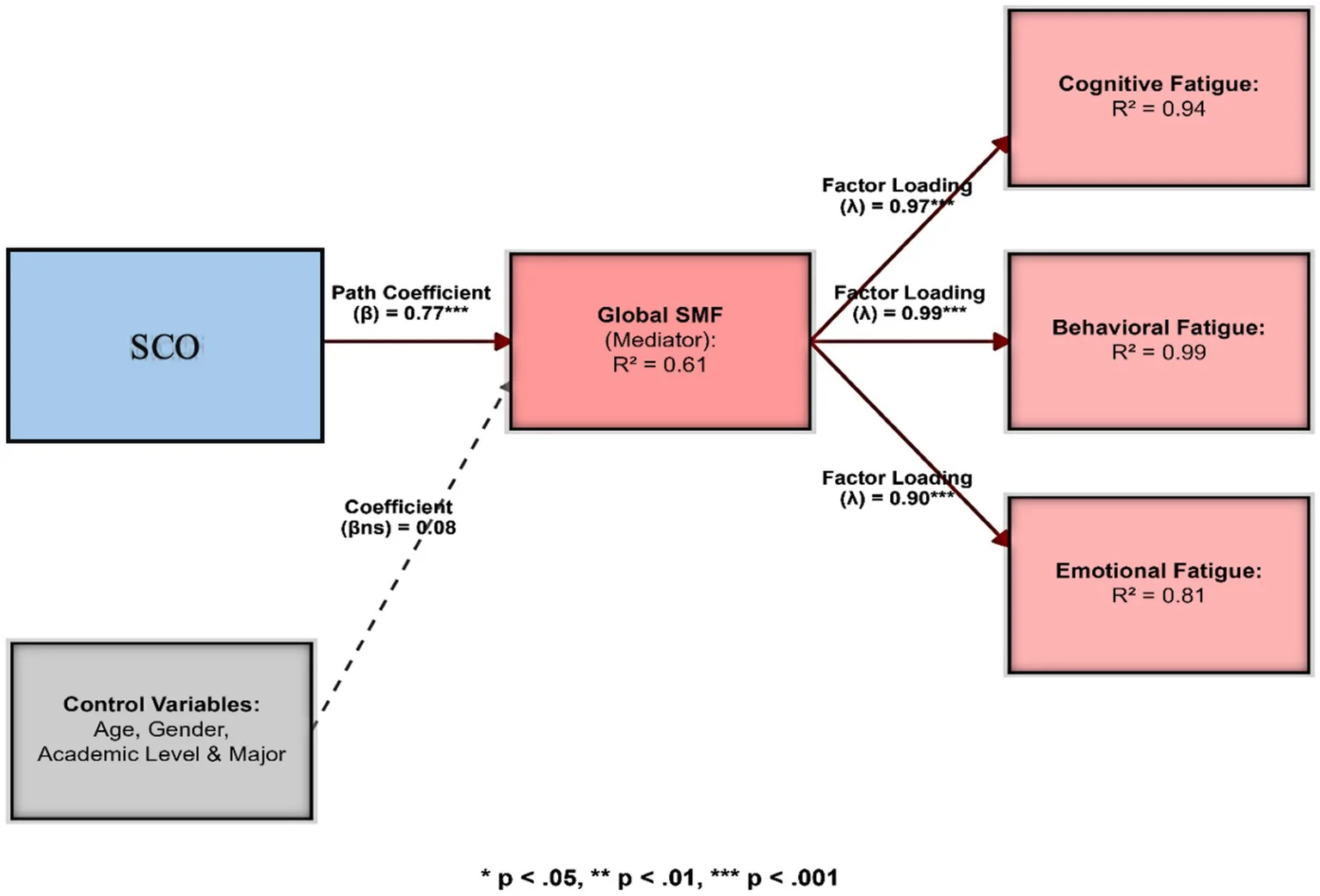
Higher-Order Structural Equation Model illustrating the association between Social Comparison Orientation (SCO) and the Global Social Media Fatigue Factor and its subdimensions.

**Table 6:** Results of the higher-order structural equation modeling.

Structural path	B	SE	β	*p*-value
Main effect				
SCO → Global SMF	0.81	0.13	0.77	< 0.001**
Control variables				
Age → Global SMF	−0.007	0.008	−0.030	0.367
Gender → Global SMF	0.065	0.040	0.062	0.105
Academic Level → Global SMF	−0.038	0.021	−0.086	0.070
Major (Natural Sci.) → Global SMF	−0.004	0.049	−0.003	0.943
Major (Health Sci.) → Global SMF	0.079	0.064	0.057	0.216
Major (Humanities) → Global SMF	0.083	0.062	0.058	0.183

*N* = 601. B is the unstandardized coefficient. SE is the Standard Error. β is the standardized coefficient. **p < 0.001. R^2^ for Global SMF = 0.607 (Explained variance for its lower-order dimensions: Cognitive R^2^ = 0.944, Behavioral R^2^ = 0.986, Emotional R^2^ = 0.806). Gender was dummy coded as Female = 1. Model Fit: CFI = 0.923, TLI = 0.916, RMSEA = 0.031, SRMR = 0.043.

These findings provide a basis for interpreting the association between social comparison orientation and fatigue-related experiences in social media contexts.

## 4 Discussion

The present study examined the association between social comparison orientation and social media fatigue among university students, drawing on Social Comparison Theory and cognitive–affective processing perspectives. The findings revealed a strong and statistically significant positive association between social comparison orientation and overall social media fatigue. This result supports the primary hypothesis and provides empirical evidence of an association between comparison-oriented processing and fatigue-related outcomes. The substantial explained variance (R^2^ = 0.607) further suggests that social comparison orientation is meaningfully associated with social media fatigue among university students. This finding is consistent with Social Comparison Theory (Festinger, 1954), which posits that individuals are inherently motivated to evaluate themselves in relation to others. In contemporary social media environments, platform dynamics amplify comparison-relevant cues, such as peer achievements, social feedback, and curated lifestyles, thereby increasing exposure to evaluative content (Verduyn et al., 2017; Kross et al., 2021). Individuals with higher comparison orientation may engage in sustained self-evaluative processing, which can impose continuous cognitive demands and contribute to fatigue-related experiences over time (Gibbons and Buunk, 1999; Buunk and Gibbons, 2007).

Importantly, the findings extend prior research by demonstrating that social comparison orientation is associated not only with isolated responses but with a broader, multidimensional fatigue construct encompassing cognitive, emotional, and behavioral dimensions. Previous studies have shown that social comparison on social media is associated with negative affect, disengagement tendencies, and fatigue-related outcomes (Cramer et al., 2016; Jabeen et al., 2023), as well as with information overload and attentional fatigue (Lee et al., 2016; Niu et al., 2022). More recent evidence further highlights the role of psychosocial stressors, such as fear of missing out (FoMO) and social comparison, in contributing to social media fatigue, suggesting that these processes operate as key internal drivers of fatigue experiences within digital environments (Parvizi et al., 2026). In addition, meta-analytic findings indicate that social comparison represents a significant predictor of social media fatigue, alongside other stressors such as information overload and FoMO, reinforcing its central role within broader stimulus–organism–response frameworks (Liu et al., 2025).

Consistent with this perspective, emerging research also shows that upward forms of comparison, particularly appearance-based comparisons, can intensify negative self-evaluations and contribute to increased fatigue, both directly and indirectly through mechanisms such as FoMO (Jaya et al., 2025). Moreover, qualitative evidence suggests that social media environments amplify comparison processes and digital identity construction, leading to emotional exhaustion and fatigue due to constant connectivity and information overload (Konstantopoulou and Brailas, 2025).

Taken together, these findings support the conceptualization of social media fatigue as a unified yet multidimensional psychological state that may be associated with sustained comparison-oriented processing. The present study builds on this literature by integrating these processes within a higher-order structural framework, thereby providing a more comprehensive account of how comparison orientation is associated with fatigue-related experiences.

Although the present study ultimately evaluated the association at the level of the higher-order social media fatigue construct, the overall pattern of findings is consistent with a higher-order conceptualization of social media fatigue. These findings suggest that the three dimensions represent closely related aspects of a common underlying construct while remaining theoretically meaningful components of the broader social media fatigue experience.

The higher-order conceptualization provides a useful framework for interpreting the observed association between social comparison orientation and social media fatigue. From cognitive–affective and cognitive load perspectives, sustained evaluative demands have been theoretically linked to cognitive depletion, emotional exhaustion, and behavioral disengagement (Eppler and Mengis, 2004; Lee et al., 2016; Hockey, 2013; Bright et al., 2015; Dhir et al., 2018; Tandon et al., 2024). These theoretical perspectives provide a plausible framework for understanding the observed association, while the present findings specifically support an association between social comparison orientation and the broader multidimensional experience of social media fatigue.

Notably, the higher-order modeling results were consistent with conceptualizing social media fatigue as a unified construct with multiple interrelated dimensions. Because the correlated three-factor and higher-order models demonstrated equivalent fit, the present data do not distinguish between these representations. Nevertheless, the higher-order model was retained because it is consistent with the theoretical conceptualization of social media fatigue as an integrated construct. The strong intercorrelations among cognitive, emotional, and behavioral fatigue suggest that these experiences tend to co-occur within the context of social media use. This interpretation aligns with prior conceptualizations of fatigue as an integrated psychological state associated with cumulative cognitive–affective demands (Whelan et al., 2020; Wang et al., 2025).

Another important finding is that none of the demographic covariates (age, gender, academic level, and academic major) showed significant direct associations with social media fatigue in the present model. After controlling for these demographic characteristics, social comparison orientation remained significantly associated with social media fatigue. The present findings should also be interpreted within the Saudi cultural context. Saudi society places considerable emphasis on social relationships, family connectedness, and social evaluation, which may increase the salience of comparison-related processes in digital environments. In such contexts, social media may offer frequent opportunities for self-evaluation relative to peers, potentially strengthening the observed association between a social comparison orientation and social media fatigue. Nevertheless, further cross-cultural research is needed to determine whether similar patterns emerge across different cultural settings.

Overall, the present study contributes to the growing body of literature on social media fatigue by integrating social comparison processes with a multidimensional conceptualization of fatigue within social media environments. Unlike prior research that conceptualized social comparison as a situational behavior, the present study conceptualizes social comparison orientation as a relatively stable processing tendency. By conceptualizing social comparison orientation as a relatively stable cognitive–affective processing tendency, the findings highlight the importance of individual differences in shaping users’ experiences in digital contexts. These insights provide a more nuanced understanding of how social information may be associated with fatigue-related outcomes beyond simple exposure or usage intensity.

## 5 Limitations and future directions

Despite its contributions, the present study has several limitations that should be considered when interpreting the findings. First, the use of a cross-sectional design limits the ability to draw causal inferences regarding the association between social comparison orientation and social media fatigue. Although the findings provide evidence of a significant relationship, future research using longitudinal or experimental designs is needed to examine the temporal dynamics and potential directional pathways underlying this association.

Second, the study relied on self-report measures, which may be subject to common method bias and social desirability effects. Although Harman’s single-factor test suggested that common method bias was unlikely to substantially influence the findings, all variables were collected using the same self-report questionnaire at a single time point. Therefore, residual common method effects cannot be completely ruled out. Future studies may benefit from incorporating multi-method approaches, such as behavioral tracking data or experimental exposure to social media content, as well as additional statistical approaches, to provide a more comprehensive assessment of comparison-related processing in digital environments.

Third, the sample consisted of university students recruited through convenience sampling, which may limit the generalizability of the findings to other populations. Social media use patterns and comparison tendencies may vary across age groups, cultural contexts, and occupational backgrounds. Future research is encouraged to replicate these findings using more diverse and representative samples, including non-student populations and cross-cultural comparisons.

Fourth, although the measurement model supported the proposed higher-order structure, several measurement-related limitations should be acknowledged. Specifically, some lower-order dimensions demonstrated relatively modest convergent validity, as reflected in the Average Variance Extracted (AVE), and the high intercorrelations among the cognitive, emotional, and behavioral dimensions suggest the need for further examination of their empirical distinctiveness. In addition, although the Arabic versions of the measures demonstrated acceptable psychometric properties in the present sample, further validation across different populations and cultural contexts would strengthen confidence in their generalizability. Furthermore, the exclusion of two reverse-worded INCOM items may limit direct psychometric comparability with studies using the original 11-item scale. Future studies may also explore alternative measurement approaches, including bifactor models or item-level refinements, to further evaluate the multidimensional structure of social media fatigue.

Finally, although the present study focused on social comparison orientation as a key individual difference and controlled for demographic characteristics, it did not examine several additional psychological and social media-related variables that may influence the observed association with social media fatigue. These include potential mediating or moderating factors such as emotional regulation, self-esteem, digital identity processes, and coping strategies, as well as social media use frequency, time spent on social media, platform type, number of platforms used, problematic social media use, information overload, and fear of missing out (FoMO). Consequently, the observed association between social comparison orientation and social media fatigue should be interpreted in the context of these unmeasured factors. Future research is encouraged to incorporate these variables to better examine their relative and combined contributions to social media fatigue.

Taken together, these limitations highlight important directions for future research to further advance understanding of how social media environments interact with individual cognitive–affective tendencies to shape psychological experiences.

## Data Availability

The raw data supporting the conclusions of this article will be made available by the authors, without undue reservation.
